# The ladies trial: laparoscopic peritoneal lavage or resection for purulent peritonitis^A ^and Hartmann's procedure or resection with primary anastomosis for purulent or faecal peritonitis^B ^in perforated diverticulitis (NTR2037)

**DOI:** 10.1186/1471-2482-10-29

**Published:** 2010-10-18

**Authors:** Hilko A Swank, Jefrey Vermeulen, Johan F Lange, Irene M Mulder, Joost AB van der Hoeven, Laurents PS Stassen, Rogier MPH Crolla, Meindert N Sosef, Simon W Nienhuijs, Robbert JI Bosker, Maarten J Boom, Philip M Kruyt, Dingeman J Swank, Willem H Steup, Eelco JR de Graaf, Wibo F Weidema, Robert EGJM Pierik, Hubert A Prins, Hein BAC Stockmann, Rob AEM Tollenaar, Bart A van Wagensveld, Peter-Paul LO Coene, Gerrit D Slooter, Esther CJ Consten, Eino B van Duijn, Michael F Gerhards, Anton GM Hoofwijk, Thomas M Karsten, Peter A Neijenhuis, Charlotte FJM Blanken-Peeters, Huib A Cense, Guido HH Mannaerts, Sjoerd C Bruin, Quirijn AJ Eijsbouts, Marinus J Wiezer, Eric J Hazebroek, Anna AW van Geloven, John K Maring, André JL D'Hoore, Alex Kartheuser, Christophe Remue, Helma MU van Grevenstein, Joop LM Konsten, Donald L van der Peet, Marc JPM Govaert, Alexander F Engel, Johannes B Reitsma, Willem A Bemelman

**Affiliations:** 1 Department of Surgery, Academic Medical Centre, Amsterdam, The Netherlands; 2 Department of Surgery, Erasmus Medical Centre, Rotterdam, The Netherlands; 3 Department of Surgery, Albert Schweitzer Hospital, Dordrecht, The Netherlands; 4 Department of Surgery, Academic Hospital Maastricht, Maastricht, The Netherlands; 5 Department of Surgery, Amphia Hospital, Breda, The Netherlands; 6 Department of Surgery, Atrium Medical Centre Parkstad, Heerlen, The Netherlands; 7 Department of Surgery, Catharina Hospital, Eindhoven, The Netherlands; 8 Department of Surgery, Deventer Hospital, Deventer, The Netherlands; 9 Department of Surgery, Flevo Hospital, Almere, The Netherlands; 10 Department of Surgery, Gelderse Vallei Hospital, Ede, The Netherlands; 11 Department of Surgery, Groene Hart Hospital, Gouda, The Netherlands; 12 Department of Surgery, Haga Hospital, The Hague, The Netherlands; 13 Department of Surgery, IJsselland Hospital, Capelle aan den IJssel, The Netherlands; 14 Department of Surgery, Ikazia Hospital, Rotterdam, The Netherlands; 15 Department of Surgery, Isala Hospital, Zwolle, The Netherlands; 16 Department of Surgery, Jeroen Bosch Hospital, 's-Hertogenbosch, The Netherlands; 17 Department of Surgery, Kennemer Hospital, Haarlem, The Netherlands; 18 Department of Surgery, Leiden University Medical Centre, Leiden, The Netherlands; 19 Department of Surgery, St. Lucas Andreas Hospital, Amsterdam, The Netherlands; 20 Department of Surgery, Maasstad Hospital, Rotterdam, The Netherlands; 21 Department of Surgery, Máxima Medical Centre, Eindhoven, The Netherlands; 22 Department of Surgery, Medical Spectrum Twente, Enschede, The Netherlands; 23 Department of Surgery, Meander Hospital, Amersfoort, The Netherlands; 24 Department of Surgery, Onze Lieve Vrouwe Hospital, Amsterdam, The Netherlands; 25 Department of Surgery, Orbis Medical Centre, Sittard, The Netherlands; 26 Department of Surgery, Reinier de Graaf Hospital, Delft, The Netherlands; 27 Department of Surgery, Rijnland Ziekenhuis, Leiderdorp and Alphen aan den Rijn, The Netherlands; 28 Department of Surgery, Rijnstate Hospital, Arnhem, The Netherlands; 29 Department of Surgery, Rode Kruis Hospital, Beverwijk, The Netherlands; 30 Department of Surgery, St. Franciscus Hospital, Rotterdam, The Netherlands; 31 Department of Surgery, Slotervaart Hospital, Amsterdam, The Netherlands; 32 Department of Surgery, Spaarne Hospital, Hoofddorp, The Netherlands; 33 Department of Surgery, St. Antonius Hospital, Nieuwegein, The Netherlands; 34 Department of Surgery, Tergooi Hospitals, Hilversum and Blaricum, The Netherlands; 35 Department of Surgery, Twee Steden Hospital, Tilburg and Waalwijk, The Netherlands; 36 Department of Surgery, University Hospital Leuven, Leuven, Belgium; 37 Department of Surgery, University Hospital St.-Luc, Brussels, Belgium; 38 Department of Surgery, Utrecht University Medical Centre, Utrecht, The Netherlands; 39 Department of Surgery, Vie Curi Medical Centre, Venlo and Venray, The Netherlands; 40 Department of Surgery, Free University Medical Centre, Amsterdam, The Netherlands; 41 Department of Surgery, Westfries Hospital, Hoorn, The Netherlands; 42 Department of Surgery, Zaans Medical Centre, Zaandam, The Netherlands; 43 Department of Biostatistics and Epidemiology, Academic Medical Centre, Amsterdam, The Netherlands

## Abstract

**Background:**

Recently, excellent results are reported on laparoscopic lavage in patients with purulent perforated diverticulitis as an alternative for sigmoidectomy and ostomy.

The objective of this study is to determine whether LaparOscopic LAvage and drainage is a safe and effective treatment for patients with purulent peritonitis (LOLA-arm) and to determine the optimal resectional strategy in patients with a purulent or faecal peritonitis (DIVA-arm: perforated DIVerticulitis: sigmoidresection with or without Anastomosis).

**Methods/Design:**

In this multicentre randomised trial all patients with perforated diverticulitis are included. Upon laparoscopy, patients with purulent peritonitis are treated with laparoscopic lavage and drainage, Hartmann's procedure or sigmoidectomy with primary anastomosis in a ratio of 2:1:1 (LOLA-arm). Patients with faecal peritonitis will be randomised 1:1 between Hartmann's procedure and resection with primary anastomosis (DIVA-arm). The primary combined endpoint of the LOLA-arm is major morbidity and mortality. A sample size of 132:66:66 patients will be able to detect a difference in the primary endpoint from 25% in resectional groups compared to 10% in the laparoscopic lavage group (two sided alpha = 5%, power = 90%). Endpoint of the DIVA-arm is stoma free survival one year after initial surgery. In this arm 212 patients are needed to significantly demonstrate a difference of 30% (log rank test two sided alpha = 5% and power = 90%) in favour of the patients with resection with primary anastomosis. Secondary endpoints for both arms are the number of days alive and outside the hospital, health related quality of life, health care utilisation and associated costs.

**Discussion:**

The Ladies trial is a nationwide multicentre randomised trial on perforated diverticulitis that will provide evidence on the merits of laparoscopic lavage and drainage for purulent generalised peritonitis and on the optimal resectional strategy for both purulent and faecal generalised peritonitis.

**Trial registration:**

Nederlands Trial Register NTR2037

## Background

Diverticular disease is an important condition in terms of healthcare utilisation and it is one of the five most costly gastrointestinal disorders in westernised countries[1]. Despite this high prevalence, treatment of all different stages of diverticular disease is still hardly evidence based, hence containing a lot of controversies.

Perforated diverticulitis is a perforation of an inflamed diverticulum of the large bowel, mostly the sigmoid, resulting in either purulent or faecal peritonitis (Hinchey stadia III or IV). Both conditions require emergency surgery[2,3]. Regardless of selected strategy emergency operations for acute perforated diverticulitis are associated with substantial morbidity (up to 50%) and mortality (15 to 25%)[3-8]. Primary sigmoidectomy with or without anastomosis has become the standard practice for patients with generalised peritonitis complicating diverticulitis [6-10] and for many surgeons the Hartmann's procedure remains the favoured option. Restoration of bowel continuity after this procedure is a technically difficult operation, with high morbidity and mortality rates[11,12]. Therefore stoma reversal after HP is only performed in 50 to 60% of the patients, thereby compromising quality of life and increasing costs[13,14].

Recently laparoscopic lavage (LL) emerged as an effective alternative for patients with perforated diverticulitis with purulent peritonitis[15]. This nonresectional procedure has first been described by O'Sullivan in 1996[16]. In 2009, a systematic review on all studies on laparoscopic lavage with a total number of 231 patients was performed. Mortality was less than 2% and a (permanent) colostoma was avoided in the majority of these patients[15-22]. So laparoscopic lavage for perforated purulent diverticulitis has a great potential in improving health and reducing costs.

Nevertheless, since sigmoidectomy is still considered the standard of care for perforated diverticulitis, implementation of LL might be variable. Some surgeons will embrace lavage because of its technical simplicity; other might be reluctant fearing failure of this novel strategy. Only a head to head comparison of both surgical strategies will provide an evidence based surgical approach of patients with perforated diverticulitis with purulent peritonitis (LOLA-arm).

In case of faecal peritonitis there is no evidence that LL is a valid alternative for a resectional strategy. But again, the optimal surgical treatment is still a matter of debate. The available literature suggests equality of Hartmann's procedure (HP) and resection with primary anastomosis (PA) regarding postoperative mortality and morbidity[5,8,9,23,24]. The likelihood of stoma closure seems higher after PA with ileostomy (85%) compared to HP (60%), but robust evidence is lacking[13,25]. Therefore, HP and PA are compared to determine the optimal resectional treatment for perforated diverticulitis with generalised purulent or faecal peritonitis, regarding stoma free survival (DIVA-arm).

### Study objectives

For this two-armed randomised trial two objectives can be defined to determine the optimal strategy for the treatment of perforated diverticulitis. First, is laparoscopic lavage for patients with purulent peritonitis superior compared to sigmoidectomy, in terms of mortality, morbidity, quality of life, health care utilisation and associated costs (LOLA-arm)? Secondly, is HP or PA the superior approach for patients with purulent or faecal generalised peritonitis in terms of stoma free survival, quality of life and cost-effectiveness (DIVA-arm)?

## Methods/Design

The Ladies trial is designed as a nationwide multicentre randomised trial in which patients with generalised peritonitis caused by perforated diverticulitis are randomised to undergo either laparoscopic lavage and drainage or resectional surgery by laparotomy.

Patients presenting with clinical signs of diverticulitis with diffuse peritonitis can be included upon the finding of free gas on plain abdominal radiography, upon the finding of free gas on CT, or upon the finding of peritonitis with diffuse fluid or gas on CT. Exclusion criteria include dementia, pelvic irradiation, steroid treatment, prior sigmoidectomy and preoperative shock with inotropic requirement. All patients need to fulfil the selection criteria and will need to give written informed consent.

**Figure 1:**
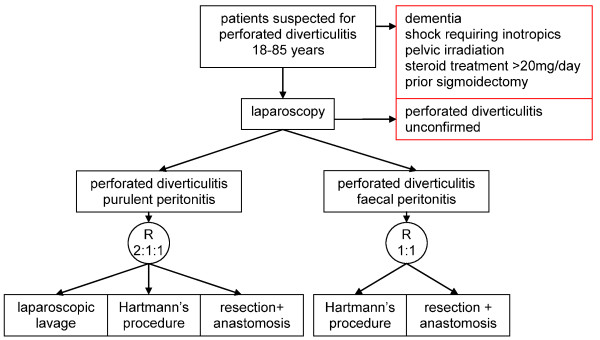
**Study profile**.

Eligible patients undergo diagnostic laparoscopy to exclude other causes of generalised peritonitis. If the diagnosis perforated diverticulitis is confirmed, the patient can be enrolled and randomised. Block-randomisation is performed during laparoscopy via the trial website according to Figure 1.

In case of purulent peritonitis (Hinchey III) patients are randomised to LL, HP or PA (LOLA-arm). The best evidence indicates that the latter two resectional strategies are equal in terms of postoperative morbidity and mortality in case of generalised peritonitis[8]. For this reason a three way 2:1:1 randomisation is performed. In case of an overt perforation with faecal peritonitis (Hinchey IV) patients will undergo laparotomy and are randomised 1:1 to either undergo HP or PA.

Patients who are either ineligible for trial entry, who show other causes of peritonitis than diverticulitis at laparoscopy or who do not wish to take part in the study are treated at the discretion of the surgeon on call. These patients will be registrated by the trial coordinator.

### Endpoints

Primary endpoint of the LOLA-arm is the combined number of mortality and major morbidity, twelve months after initial surgery. Secondary endpoints of the LOLA-arm are quality of life, health care utilisation and associated costs. Mayor morbidity includes reintervention, fascial dehiscence, incisional hernia, myocardial infarction, urosepsis, respiratory failure and renal failure. Respiratory failure is defined as a SOFA score of less than 300. Renal failure is defined as a threefold creatinine increase or a GFR decrease over 75% or a urinary output of less than 0.3/kg/h for 24 hours or anuria for twelve hours.

Primary endpoint of the DIVA-arm is the stoma free survival within twelve months after initial surgery. Secondary endpoints are quality of life and cost-effectiveness.

### Participating centres

More than thirty-five teaching hospitals in the Netherlands are participating in this trial, including six academic centres.

### Study population

This study consists of patients eligible for surgical treatment of perforated diverticulitis. Inclusion criteria are age between 18 and 85 years, a clinical suspicion for perforated diverticulitis and free gas on plain abdominal radiography, free gas on CT, or peritonitis with diffuse fluid or gas on CT.

### Ethics

This study will be conducted in accordance with the principles of the Declaration of Helsinki and Good Clinical Practice guidelines. Medical ethics approval has been obtained by the medical ethics committee from the Academic Medical Centre in Amsterdam, dated September 30th, 2009. Prior to randomisation, written informed consent must be obtained from all patients.

### Study outline

Diagnostic laparoscopy: a careful inspection of the stomach, duodenum and sigmoid is performed to localise the site of perforation. In case of peritonitis due to a perforated diverticulum it must be attempted gently to locate the site of perforation. Careful removal of adherent omentum or bowel is tried. If clearly adherent, it should be left in place.

If no obvious perforation is apparent and faecal content is absent, the patient is randomised online between treatment with LL, HP or PA in a ratio 2:1:1.

In case of an overt perforation or intra-abdominal contamination with faeces, the patient is not eligible for LL and is randomised between HP and PA.

LL: the abdominal cavity is irrigated with six litres of warm saline. At the end of the procedure a Douglas drain is inserted via the right lateral port.

HP: The perforated diseased part must be resected. There is no need of having the distal transsection line on the proximal rectum. An end-colostomy is performed according to the preference of the operating surgeon, the same accounts for closing the rectal stump.

PA: Sigmoidectomy is done according to the guidelines of the American Society of Colon and Rectal Surgeons[26,27]. The distal transsection margin has to be on the proximal rectum, the proximal margin is determined by the absence of wall thickening due to diverticulitis. The type of anastomosis and the decision to perform a defunctioning loop-ileostomy are to the discretion of the surgeon on call.

Leaving a Douglas drain after resectional surgery is at the discretion of the operating surgeon. The resected tissue is sent for histological investigation to exclude malignancy.

Antibiotics are administered for seven days in both groups. Postoperatively, oral diet and mobilisation are advanced as soon as possible. Within four to six weeks after surgery a sigmoidoscopy is performed to exclude malignancy as the underlying cause of the perforation.

After the sigmoidoscopy is performed, the patient will be offered reversal of the stoma, when he or she is found eligible for surgery by the surgeon and anaesthesiologist.

### Statistical analysis

The analysis will be performed in accordance with the intention to treat principle.

In the LOLA-arm of the study, the assumpted difference in the combined number of mortality and major morbidity between laparoscopic lavage and resection is 15%. With a two sided likelihood ratio test and a significance level of 0.05, a sample size of 132:66:66 will be necessary to detect this difference. With a group size of a hundred patients per arm it is also possible to find a significant difference (alpha = 0.05, beta = 0.1) of at least 10% in subscales of the SF-36, a validated quality of life questionnaire, at two, four, thirteen, 26 and 52 weeks after initial surgery.

In the DIVA-arm 212 patients are needed to significantly demonstrate a difference in stoma free survival between both treatment arms, using log rank statistics with a power of 90% and a type I error of 5%. The suspected postoperative mortality for HP and PA is equally high (+ 15%)[8]. About 60% of the patients that underwent HP have their stoma reversed[11,12]. When corrected for the expected mortality before reversal, the reversal rate will be 50%. Patients with a protective loop-ileostomy after PA will have their enterostomy reversed in over 85%[12]. After correction for expected mortality before reversal, this will result in a 72% stoma reversal rate in the initial patient population.

### Economic evaluation

Comparisons of the different surgical strategies in the economic evaluation will be analogous to the analyses of the clinical endpoints. The economic evaluation will be performed from a societal perspective, with the costs per unit improvement on the primary clinical endpoints, defined as combined mortality and morbidity for the LOLA-arm, and stoma free survival for the DIVA-arm.

We hypothesise that a more effective intervention will be associated with less health care utilisation as well as absence from paid work (productivity costs). Therefore, the primary analysis will be a cost-effectiveness analysis that evaluates costs associated with an improved surgical outcome.

In addition, a secondary analysis will evaluate cost differences in relation to differences in quality-adjusted life-years (QALYs). This cost-utility analysis, resulting in an incremental cost-effectiveness ratio expressed in costs per QALY, will be included to allow comparison with other health-related interventions or programs. With a study horizon of twelve months, no discounting will be applied. We will differentiate between direct medical, direct non-medical and indirect costs.

### Data collection and monitoring

An electronic Case Report Form (CRF) will include general patients data: sex, age, medical history, POSSUM-score, preoperative APACHE-score, surgical parameters, Hinchey score, data concerning type of intervention, complications, mortality, duration of hospital and intensive care stay and the patients response to the questionnaires.

Patients will be followed for a period of twelve months. During this follow-up period patients will complete a set of questionnaires (SF-36, EQ-5D and GIQLI) two, four, thirteen, 26 and 52 weeks after the initial surgery. The questionnaires will be sent to the patients by mail accompanied by a stamped return envelope. Collection of the questionnaires will be safeguarded by the trial coordinator.

At four, thirteen, 26, 39 and 52 weeks after initial surgery, the patients will be asked to complete questionnaires to assess complications, additional interventions, readmissions, duration of hospital and intensive care stay, visits to the outpatient clinic, number of days of sick leave and to ensure completions of the questionnaires.

### Patient safety

An independent data monitoring and safety committee has been established to interpret the data from the current trial, to monitor any early significant differences between the groups of treatment and to make interim analyses to decide on continuation of the study after every 25 included patients.

An independent trial monitor will monitor the study procedure and the data of included patients.

A data management agency created the online database of the study to guard the entry of data by the local investigators. The same organisation has trained all trial coordinators, all local investigators and some local co-investigators on the guidelines of Good Clinical Practice.

The trial coordinators have trained all other personnel on the protocol, on asking informed consent, on reporting Serious Adverse Events and on data entry.

According to the Good Clinical Practice guidelines, a list of Serious Adverse Events is defined. All events on this list have to be reported by the local investigators to the trial coordinators within 24 hours after the event. These events will be reported to the central Medical Ethics Committee (CCMO) within 24 hours afterwards. With this measure, the central Medical Ethics Committee compares the incidence of complications between the arms of the trial and can decide on continuation of the trial.

## Discussion

Since the introduction of laparoscopic lavage and drainage for purulent peritonitis for perforated diverticulitis in 1996, the number of patients treated with this new method had gradually inclined. However, there have been no publications of high methodological quality on this topic[28]. Therefore we do not know whether laparoscopic lavage is in fact a safe and effective treatment. Since the existing publications do promise a significant reduction in mortality and major morbidity, a randomised trial is appropriately warranted. A data monitoring committee will guard the methodological quality of the study, the safety of the patients, and monitor any early significant differences between the different surgical strategies.

We have not found any evidence that laparoscopic lavage is a safe treatment for perforated faecal peritonitis. Therefore in this group of patients randomisation will only take place between the two resectional strategies.

In the presented study all patients suspected for perforated diverticulitis are included, and a midline laparotomy can be avoided in selected patients with other pathology.

We do not know whether the lavage itself is important for the treatment of the peritonitis, since there are no publications on the treatment of purulent perforated diverticulitis with diagnostic laparoscopy and antibiotic treatment alone. Laparoscopic lavage in combination with antibiotic treatment however, has been examined in a systematic review with very promising results[28].

The stoma reversal rate is the primary endpoint for the DIVA-arm of the trial. Questions could be raised about the benefits of this reversal for a patient that is incontinent for faeces. A definitive colostoma for this specific group of patients might be preferable considering daily care. However this group of patients will be small and no studies have compared quality of life for incontinent patients with or without a stoma. The colostoma and ileostoma show equal impact on the patients quality of life, [29] and quantification of incontinence problems is unpractical in the emergency setting. Therefore incontinence is not established as an exclusion criterion. All resections will be performed with the intention of stoma reversal.

In the Netherlands the standard of care for perforated diverticulitis is either HP or PA. Resection with primary anastomosis is a type of treatment not mastered by every gastrointestinal surgeon. In the emergency setting, some surgeons might prefer HP, fearing anastomotic leakage. However, there is no clear evidence available showing a difference in mortality and major morbidity between HP and PA. Therefore we decided to include treatment with PA in the randomisation process of the LOLA-arm as well.

Our hypothesis is that PA leads to a 22% higher stoma free survival, and that this procedure might be advocated as the new standard of care in selected patients with generalised peritonitis caused by perforated diverticulitis.

## List of abbreviations

LOLA-arm: Laparoscopic lavage and drainage or sigmoidectomy with HP or PA for purulent peritonitis for perforated diverticulitis; DIVA-arm: Sigmoidectomy with HP or PA for generalised peritonitis for perforated diverticulitis; SF-36: Quality of Life Questionnaire Short Form 36; GIQLI: Gastro Intestinal Quality of Life Index; EQ-5D: Euro Quality of Life Questionnaire; LL: Laparoscopic lavage; HP: Hartmann's procedure; PA: Sigmoidectomy with primary anastomosis.

## Competing interests

The authors declare that they have no competing interests.

## Authors' contributions

HS drafted the manuscript. WB, JL and JV co-authored the writing of the manuscript. All other authors participated in the design of the study during several meetings and are local investigators at the participating centres. All authors edited the manuscript and read and approved the final manuscript. The design of the Ladies trial has been approved by the members of the Dutch Diverticular Disease Collaborative Study Group.

## Pre-publication history

The pre-publication history for this paper can be accessed here:

http://www.biomedcentral.com/1471-2482/10/29/prepub
